# Risk of severe postpartum episodes

**DOI:** 10.1192/j.eurpsy.2021.202

**Published:** 2021-08-13

**Authors:** A. Wieck

**Affiliations:** Greater Manchester Mental Health Nhs Foundation Trust, University of Manchester, Manchester, United Kingdom

## Abstract

**Abstract Body:**

The risk of mothers to develop a severe mental illness is dramatically increased in the first three months after giving birth. Childbirth has the strongest relationship with postpartum affective psychosis, a condition that is characterized by an acute onset of florid symptoms, usually within 2 weeks of delivery, and atypical features, such as rapidly fluctuating psychotic symptoms, florid motor symptoms, perplexity and high risks to the mother and her baby. Follow up data of women with a first episode suggest that some women only become ill in the context of childbirth whereas in others it is an expression of a lifelong bipolar disorder. Whether this reflects two distinct forms of the disorder or different degrees of vulnerability requires future study. The profound hormonal and metabolic as well as psychosocial changes in the perinatal period give rise to a number of hypotheses that seek to explain the pathogenesis of postpartum psychosis. Current research findings on biological and psychosocial risk factors will be discussed as well as what is currently known about responses to treatment.

**Disclosure:**

No significant relationships.

